# Four-Dimensional Bioprinting As a New Era for Tissue Engineering and Regenerative Medicine

**DOI:** 10.3389/fbioe.2017.00061

**Published:** 2017-10-17

**Authors:** Pedro Morouço, Wanda Lattanzi, Nuno Alves

**Affiliations:** ^1^Centre for Rapid and Sustainable Product Development, Polytechnic Institute of Leiria, Marinha Grande, Portugal; ^2^Institute of Anatomy and Cell Biology, Università Cattolica del Sacro Cuore, Rome, Italy

**Keywords:** stimuli–responsive biomaterials, four-dimensional bioprinting, bioinks, bioprocesses, bioreactors

In the era of “big data,” coping with a society that is in constant development, the discovery of “new” scientific and technological knowledge must (i) progress at an incredibly fast pace, (ii) target a wide audience, and (iii) have a practical impact in the society by addressing relevant challenges. The health sciences are naturally a priority area of research, mostly because of the impact they have on augmenting human life expectancy and improving well-being, by developing advanced tailored approaches to address patient-specific needs. As an example, during the past decade, the wide amount of data gathered from the human genome project, along with the improved knowledge of genome regulatory mechanisms, brought about the development of synthetic biology and genomic editing techniques (Singh et al., 2017). This massive advancement has been contributing not only to a better definition of disease mechanisms, but, importantly, also to the development of personalized therapeutic approaches. Indeed, the so-called “precision medicine” represents one of the main themes addressed by the European Commission health program, being featured in several distinct topics in the Horizon 2020 research and innovation program.^1^

In this context of incessant development of tools and improvements in the biomedical field, tissue engineering is playing a leading role as a multidisciplinary research branch. The ambition to cope with the complexity of human tissues, aimed at regenerating those hampered by diseases and age-related degeneration, has been the major goal of tissue engineering, which emerged in the 1980s, as a frontier scientific field with an enormous potential. An overwhelming amount of tissue engineering strategies have been developed since then, aimed at regenerating bone, cartilage, skin, and many other tissues and organs, in the attempt to bridge structure (gross anatomy and histological architecture) with the corresponding function (physiology and cell biology), as a paramount challenge to be solved (Campana et al., 2014). On this regard, several efforts have been made worldwide to develop synthetic or semisynthetic constructs that could mimic native tissues.

Most of the human native tissues are made of complex three-dimensional (3D) structures, presenting different shapes, architectures, specific cell types, and extracellular matrix compositions. Furthermore, these tissues are extremely plastic and not static, having unique functions suitable to dynamic changes in tissue conformations. Thus, the conventional approaches of creating static 3D structures are not sufficient for its usage in biomedicine and the achievement of 3D complex organ structures is far from being tangible (Woodfield et al., 2017). Implants for tissue engineering strongly depend on the (bio)materials and the manufacturing process. The conventional manufacturing processes do not present a properly control over pore size, geometry, and spatial distribution, not guaranteeing pore interconnectivity; which are key features for successful tissue regeneration (Hollister, 2005). Therefore, additive manufacturing (also known as 3D printing) techniques have gained an increased importance for the scientific community overlapping the referred drawbacks. The usage of computer-aided processes for patterning and assembling living and non-living materials with a prescribed 2D or 3D organization promoted bioprinting to produce bioengineered structures serving in regenerative medicine, pharmacokinetic, and basic cell biology studies (Guillemot et al., 2010). Bioink-based 3D bioprinting technologies are being employed to engineer experimental models of tissue and organ substitutes (Ji and Guvendiren, 2017). Some examples are full-thickness skin models, retaining all characteristics of human skin, and allowing to restore even large defects (Cubo et al., 2016; Pedde et al., 2017; Pourchet et al., 2017); vascular structures, such as vessels and heart valves (Datta et al., 2017); bone and osteochondral constructs (Yang et al., 2017); mini-liver with partial functional properties (Zhong et al., 2016); neural structures, among a growing number of other biological structures (Cui et al., 2017; Li et al., 2016a).

Nevertheless, 3D bioprinting has been focused on the development of constructs that lack a crucial element for appropriately mimicking native live tissues: the ability to acutely change according to functional status and changes in the environment (Khademhosseini and Langer, 2016). That is why leading research groups have recently proposed the four-dimensional (4D) bioprinting as an enhanced approach for tissue engineering and regenerative medicine. 4D bioprinting aims to include the ability of promoting dynamic changes of the structure, improving the functional response of the construct. It requires stimuli–responsive biomaterials that should be developed, suitable to be utilized in optimized bioprinting equipment, leading to constructs that are biologically active and can change their physical–chemical properties using the designed stimulation.

These features are vital to maintain long-term function of biosynthetic constructs after implantation, maintaining cellular homeostasis, self-renew, respond to tissue mechanical and chemical stimuli, and integrate with host cells and tissues. To this aim, the implementation of living cells, possibly of different lineages, enable to mimic the niche microenvironment (Cui et al., 2017). On this regard, somatic multipotent stromal cells, isolated from different postnatal organs and tissues (including bone marrow, adipose tissue, amniotic membrane, among others), represent a promising source, as they grant relatively high viability and yield, limited harvesting-associated morbidity, robustness and mechanical resilience, limited immunogenicity, and extended trophic properties. Most of their functional features are believed to reside in their paracrine functions: once implanted they release bioactive molecules such as angiogenic factors and immunomodulatory cytokines that are actively involved in tissue regeneration (Barba et al., 2017; Silini et al., 2017). By offering the potentiality to generate synthetic tissues and organ subunits *in vitro* relying on adult stem cells, bioprinting may allow overcoming the ethical burden associated with the development of either xenogeneic or embryo-derived engineered constructs to restore damaged human organs (Pedersen et al., 2012).

Still, several challenges arise. For instance, bioinks must be optimized to achieve successful bioprinting and processes must be mechanically designed to obtain robust shape-changing capability of the constructs (Li et al., 2016b). Additionally, we consider that specific bioreactors for complex tissue function maturation need to be invented and evaluation procedures should be defined to examine the functionality response. Only gathering these four issues, we will be able to design and produce reliable structures able to experience dynamic changes according to the changes provoked by the environment. Indeed, despite bioprinting being a revolutionary technology aimed at building living tissues and organs with definite cytoarchitectonics, it has not yet been translated efficiently into the clinical setting. This is mainly due to current limitations in building human-scale functional constructs, facing vascularization, and dynamic homeostatic regulations as the main operative challenges (Ji and Guvendiren, 2017; Mir and Nakamura, 2017; Ravnic et al., 2017).

Some research groups have been studying the 4D potential, mainly by the first mentioned challenge: the biomaterial. Developing “smart” biomaterials (also referred as “intelligent,” “stimuli–responsive,” “stimuli-sensitive,” or “environmentally sensitive”) to allow the dynamic changes of the structure (Furth et al., 2007) is critical for enhanced tissue engineering approaches (Gao et al., 2016). Optimally, they should have self-adaptability, self-sensing, shape-memory, responsiveness, multifunctionally, self-repair, and decision making. Shape memory polymers (SMPs) are a class of smart materials able to “memorize” a permanent shape through physical or chemical crosslinking. This allows them to be deformed and fixed temporarily by vitrification or crystallization of the polymer chain and then returned to the permanent shape by the application of an external stimulus (e.g., heat) (Liu et al., 2007).

Recent advances in SMP have enabled the study of programmable, shape-changing, cytocompatible scaffolds (Tseng et al., 2016), mostly for bone tissue engineering and using conventional manufacturing processes. For instance, porous foams triggered to recover at body temperature exhibited two-way reversible shape memory under the bias of a compressive load (Baker et al., 2013) and have shown to be cytocompatible with osteoblast-like cells (Rychter et al., 2015). Furthermore, SMP scaffolds have exhibited excellent bioactivity *in vitro*, supporting osteoblast adhesion, proliferation, and osteogenic gene expression (Zhang et al., 2014) and promising results to be used as a synthetic bone substitute in an *in vivo* non-load bearing critical-size defect model (Liu et al., 2014). Additionally, porous fibrous scaffolds by electrospinning have also been studied. Recently, even after being processed into fibrous structures, the copolymers maintained their shape memory properties, and all the fibers exhibited excellent shape recovery ratios (Kai et al., 2016). The biological assays demonstrated osteoblast proliferation, functionally enhanced biomineralization-relevant alkaline phosphatase expression and mineral deposition, corroborating previous studies using the same manufacturing technique (Bao et al., 2014). Nevertheless, the osteogenic differentiation capacity of stem cells resident in shape memory scaffolds following the programmed shape change remains unclear (Tseng et al., 2016).

The detailed knowledge of the biological events involved in tissue homeostasis and related disorders would be crucial to achieve a successful structural and functional regeneration. Specifically, we believe that the accurate characterization of stem cell niches and their local environment (Lattanzi et al., 2015), including the molecular networks orchestrating their proliferation-differentiation switch, could help tailoring bioprinting strategies to improve self-regeneration through the implementation of molecular targeted approaches. Therefore, the most promising approach is to optimize the cell-constructs interactions, becoming feasible to explore the usage of computer modeling to examine the further responses.

## Author Contributions

PM and WL conceived the idea. PM, WL, and NA have been involved in critically revising for important intellectual contents. All authors read and approved the final manuscript and agreed to be accountable for all aspects of the work.

## Conflict of Interest Statement

The authors declare that the research was conducted in the absence of any commercial or financial relationships that could be construed as a potential conflict of interest.

## Footnotes

^1^http://ec.europa.eu/research/.
